# Supplementary figures and images for: Urine Flow Cytometry Parameter Cannot Safely Predict Contamination of Urine—A Cohort Study of a Swiss Emergency Department Using Machine Learning Techniques
Source: Diagnostics (Basel). 2022 Apr 16;12(4):1008. doi: 10.3390/diagnostics12041008 (PMC9025120; doi:10.3390/diagnostics12041008)

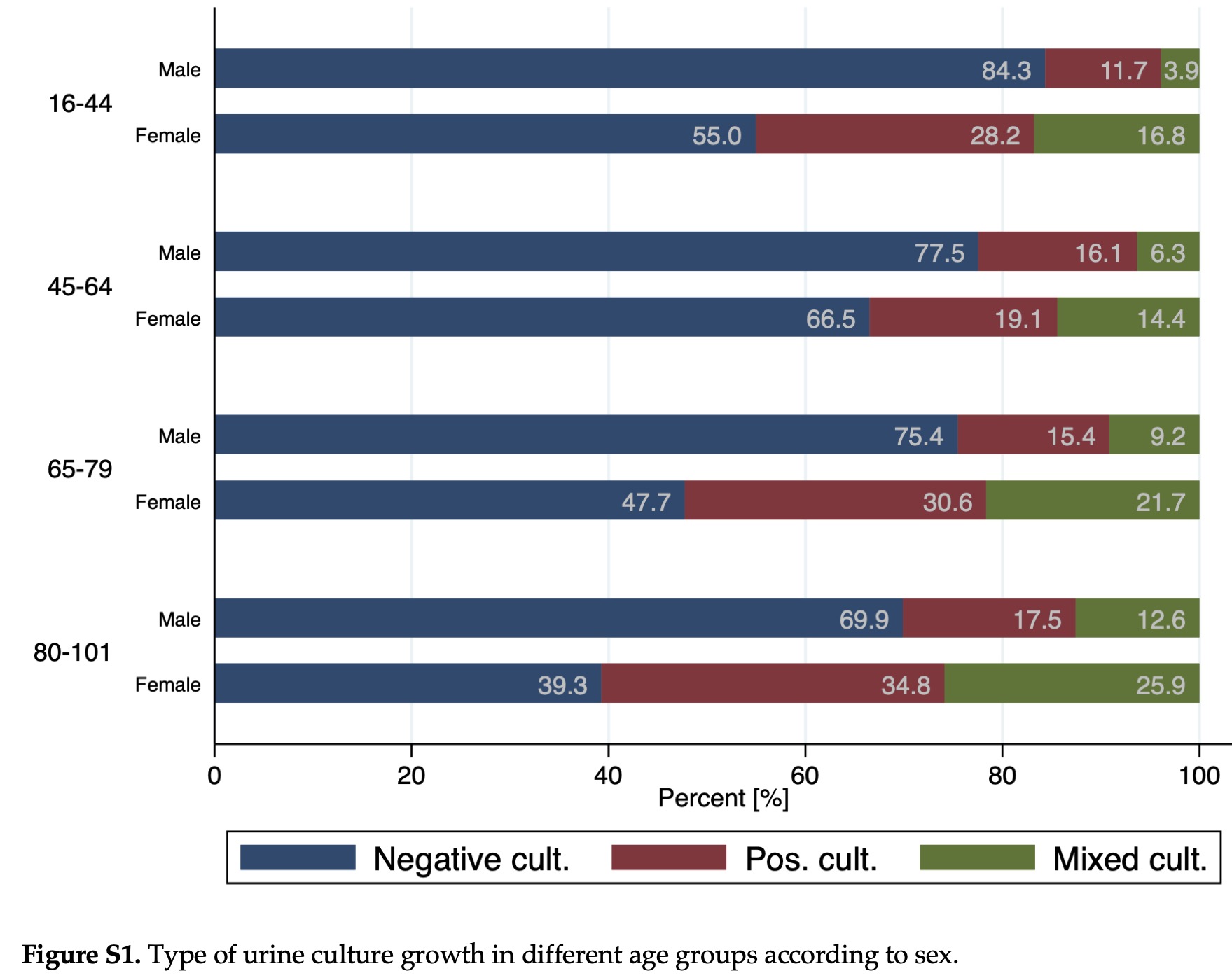

Supplement: Supplementary file 1 [file diagnostics-12-01008-s001.zip › diagnostics-1668060-supplementary.jpeg]
